# Sensitivity Analysis of Determining the Material Parameters of an Asphalt Pavement to Measurement Errors in Backcalculations

**DOI:** 10.3390/ma14040873

**Published:** 2021-02-11

**Authors:** Paweł Tutka, Roman Nagórski, Magdalena Złotowska, Marek Rudnicki

**Affiliations:** Faculty of Civil Engineering, Warsaw University of Technology, 00-637 Warsaw, Poland; romnag@il.pw.edu.pl (R.N.); m.zlotowska@il.pw.edu.pl (M.Z.); marr@il.pw.edu.pl (M.R.)

**Keywords:** road pavement mechanics, flexible pavement, backcalculation, stiffness moduli, sensitivity analysis, FWD

## Abstract

Nondestructive tests of road pavements are among the most widely used methods of pavement condition diagnostics. Deflections of road pavement under a known load are most commonly measured in such tests, e.g., with the use of falling weight deflectometer (FWD). Measured values allow to determine the material parameters of the road structure, corresponding to the obtained results, by means of backcalculations. Among the factors that impact on the quality of results is the accuracy of deflection measurement. Deflection basins with small differences of displacement values may correspond to significantly different combinations of material parameters. Taking advantage of them for mechanistic calculations of road pavement may eventually lead to incorrect estimation of the remaining fatigue life and then inadequate selection of pavement reinforcement. This study investigated the impact of measurement errors on the change of the obtained values of stiffness moduli of flexible road pavement layers. Additionally, the influence of obtained material parameters on the values of key pavement strain, and consequently on its design fatigue life was presented.

## 1. Introduction

The determining of the state of displacement, stresses, and strains of road pavement structure is essential in the process of road pavement design and diagnostics. The maximum tensile strain at the bottom of the asphalt layer and the maximum compressive strain at the top of the subgrade under standard axle load are the crucial values applied to assess the durability of flexible road pavement using mechanistic-empirical method. They are determined by adopting a certain mechanical model of the pavement structure, in which a knowledge about the values of material parameters is required. In the case of existing pavement, nondestructive tests are usually used for material parameters identification. In commonly used in situ nondestructive testing, the pavement is subjected to a known load (usually impulse load), the maximum pavement deflection in time is measured at measuring points on the upper surface. The backcalculations, based on the mechanical model of the pavement, then can be used to determine the material parameters. It should be noted that such tests are burdened with many errors, e.g., inaccurate measurement of deflections, uncertainty of load application, and inaccurate determination of the thickness of pavement layers (usually determined by testing a core sample, or also as a result of nondestructive tests). One issue that needs to be asked is whether the measurement uncertainties will influence the process of backcalculations so that the obtained values of material parameters will be subject to a large error, which may cause incorrect estimation of road pavement durability. This study aims to assess the sensitivity of backcalculations to inaccuracies of pavement deflection measurements, regardless of the measurement method adopted for these deflections.

Nondestructive tests of road pavements using the measurement of pavement deflections are widespread in the diagnosis of road pavement condition, e.g., falling weight deflectometer (FWD). Measured deflection values indicate the state of road pavement [1]. There are many parameters related to deflections which inform on the pavement condition [2]. Moreover, pavement deflection correlates with other factors indicating the pavement condition, such as pavement damage, which reflects on the level of pavement cracks [3]. Additionally, falling weight deflectometer time history data may be used in relative cracking assessment [4]. Nondestructive tests based on the measurement of deflections are supported by geo-radar tests, which enable to determine the thickness of pavement layers in a continuous manner [5]. The measurement of pavement deflection under a known load allows to calculate the material parameters of pavement layers, in particular the stiffness modulus. The backcalculations are a process requiring the application of a method of calculating pavement response, with a specific mechanical model of the pavement, including the load, determining the deflections. The procedure determines combinations of material parameter values until the deflections obtained are sufficiently like the measured values. Backcalculations are usually carried out to determine the modulus of elasticity in the linearly elastic layer half-space road structure model composed of homogeneous and isotropic layers, with known thicknesses of material layers. These moduli are interpreted as stiffness moduli, e.g., dynamic moduli of longitudinal stiffness (values of complex moduli) at a frequency corresponding to a loading speed and at the temperature of the asphalt layer during the measurement [6]. In practice, the static load model is often used [7]. Sometimes viscoelastic properties of asphalt layers are applied [7]; less frequently nonlinear models of aggregate base course, and subgrade are used [7]. The key aspect of the practical use of backcalculations is the accuracy of the obtained results. The studies demonstrate variations of moduli of all layers along the length of sections [8]. Some of these differences are caused by inaccuracies of backcalculations. Due to the variability of FWD deflection fuzzy-based approaches for the selection of a representative basin over multiple deflection basins collected for a specific section are used [9]. Factors influencing the calculation errors are described in the paper [10]. The following are distinguished here: systematic and random errors in the deflection measurement; error resulting from the presence of a stiff bottom layer (which should be detected during backcalculations); and errors resulting from nonlinear properties of the aggregate base course and subgrade, which depend on the stresses in structure. In addition, [11] draws attention to errors of the values of layer thickness, which directly affect the accuracy of backcalculations. In extreme cases, it is impossible to reliably determine the stiffness moduli for thin layers of the structure. Therefore, most often asphalt layers are not considered in the division into pavement layer, binder layer, and asphalt base course.

Calculation model which enables to determine the deflections with the assumed properties of material layers, is required to conduct backcalculations. In many programs for backcalculations, simplified models are used [7], e.g., without considering the inertia forces in the pavement, despite dynamic (impulse) loads causing the measured deflections. It should be noted that there is a possibility of more complex calculations, including dynamic effects. For example, the paper [12] uses a dynamic model considering the damping in the ground (Rayleight’s damping) and viscous damping, paper [13] additionally considers pavement roughness in dynamic calculations. The paper [14] analyzes how the obtained results of dynamic calculations are affected by the following factors: viscoelastic properties of asphalt layers, pavement temperature, loss of continuity between layers, and the presence of a stiff bottom layer (bedrock). It is important to pay attention to the effect of temperature change on the obtained results—the properties of mineral-asphalt mixture strongly depend on the temperature. In practice, temperature has a great influence on the test results and interpretation of the obtained deflection values [15]—especially large in the case of deflection measurements near of the load axis [16]. Temperature has less effect on deflection when the pavement is cracked [16]. It is worth noting that considering dynamic effects has an impact on the accuracy of the results [17]; their exclusion has influence on the results of backcalculations. Deflection results of FWD depend on the load frequency domain. In the paper [18], the method for normalizing the frequency of load and displacement functions was verified, which improved the backcalculations results. A further important issue is the way of modelling unbound mixtures. Most often they are assumed to be homogeneous, isotropic, and linear elastic, in other approaches more complex models are assumed, e.g., cross anisotropy [19]. In this paper, a dynamic calculation model was assumed due to the significant influence of d’Alembert’s inertia forces on the calculated deflections [20,21]. In addition, to avoid too many material parameters, linearly elastic models were used. With a larger number of parameters, possible errors in determining their values would be even more obvious.

The influence of measurement errors in backcalculations was outlined in the paper [22]. The impact of measurement uncertainty of deflection and thickness of layers on the calculation results in three programs conducting backcalculations was analyzed. A detailed approach to the study on the significance of error in determining the layer thickness from the results of backcalculations is presented in the paper [23]. It was described that for flexible pavement structure, in case of the error of determining the thickness of asphalt layer of ±15%, the error of determining the asphalt stiffness modulus is from −30% to 40%. For the sensitivity analysis, such calculation methods as Monte Carlo method, Fourier amplitude sensitivity test [24], or Elementary effects method [25] were also used. The paper [26] presents the analysis results of the influence of error on the results of backcalculations of stiffness moduli and then on the calculated durability of pavement. In this paper, the Monte Carlo method was applied, which consists in the randomization of the disturbances in the value of pavement deflections. Computational methods aimed at reducing the sensitivity of calculation results to errors occurring in the identification of calculation data, such as genetic algorithms [27,28,29], data mining [30], artificial neural networks [31,32], fuzzy logic [33], and conformal prediction [34], become increasingly popular.

This paper presents the dispersion of calculation results of the pavement layer stiffness moduli, key strains, and calculated durability of the pavement depending on errors in the measurement of pavement deflections, which are generated randomly using the Monte Carlo method. The dispersion measure used in this study is the coefficient of variation, i.e., the ratio of the standard deviation value to the average value of parameters. It enables to compare the variability of parameters with different orders of magnitude. The paper introduces new elements in comparison with the presented literature. In the study, the dispersion of pavement durability determined according to Mechanistic-Empirical Pavement Design Guide 2008 method was calculated; for comparison, in the study [26], the effect of error on durability was calculated using the older mechanistic-empirical method. In addition, in comparison to the paper [26], for deflection calculations, a dynamic analysis was used in the Abaqus program, using the finite element method, considering the d’Alembert inertia forces in the pavement and the speed of wave propagation—these phenomena influence the results obtained from backcalculations in the FWD study. Moreover, the introduced random generation of deflection error not only considers independent errors for each measuring device, but also a systematic error in which the measurement disturbances of individual deflections are interrelated. Such a method of error generation does not cause a “shape change” of the deflection basin. This enables the assessment of the influence of deflection basin shape on the sensitivity of backcalculations.

## 2. Materials and Methods 

### 2.1. Calculation Scheme

In this paper, backcalculations were used to determine the values of stiffness moduli of pavement layers based on pavement deflection, using the assumed calculation model of pavement and its load. In the first step, the initial values of the stiffness moduli are assumed to calculate the deflection of pavement. The deflections obtained from the calculations are compared with the deflections obtained from the measurements. In case the difference is greater than the assumed limit, the values of material parameters are changed according to the established procedure. Recalculations for the new parameter set are carried out. The process is conducted until the results for the measured and calculated deflections are consistent with the assumed level of compliance.

In this study, the maximum deflection was determined at points with a specified distance from the axis of load. The backcalculations were conducted for the pavement deflection basin, defined by seven points located at a distance from the load axis by: 0 m; 0.2 m; 0.3 m; 0.6 m; 0.9 m; 1.2 m; 1.5 m. The root mean square (RMS) was used to determine the difference between the measured and calculated deflections. The purpose of the computation was to find the minimum of this difference function. This minimum should be sufficiently close to zero—according to the established criterion for completing the calculation. The function was minimized by the secant method.

The model of flexible pavement (typical for average traffic intensity) in the form of three-layer half-space, with linear-elastic, homogeneous, isotropic layers [35] was used (two upper layers of constant thickness and a lower one of infinite thickness). The top layer (*j* = 1) is a model of asphalt layer, the middle layer (*j* = 2) is a model of unbound aggregate base course, and the third one (*j* = 3) is a model of natural subgrade. Therefore, the stiffness modulus is Young’s modulus of elasticity of layers. The Poisson’s coefficients and mass density of layers were assumed in the calculation process. Thicknesses of the layers were considered known (e.g., from the core at the measuring point). Continuity of displacements on common surfaces of layers has been assumed—physically it means full adhesion of the modeled layers.

In the case of many asphalt layers, each of them may be modelled separately which causes an increase in the calculation complexity, especially when the layers are allowed to slip over each other, but the basic steps are as presented in the paper.

The load of the pavement (on the upper surface) was distributed evenly over a circular surface with a defined variation in time, simulating the action of a device generating deflections.

The process of determining the stiffness moduli of the road pavement structural layers was iterative. In the following steps, the changes of each stiffness modulus were calculated to ensure that the obtained deflection values were similar to the measured deflections. In the first step, the stiffness moduli had the following values: 5000 MPa for the asphalt layer, 100 MPa for the aggregate base course, and 50 MPa for the subgrade. The dependence of the results on the starting point was also checked—the values of material parameters were depended to some extent on the seed moduli, the variability due to the seed moduli was about 10 times smaller than that due to analyzed measurement errors for the considered measured deflection basin. It was therefore decided to focus on the impact of measurement errors.

The following designations have been used to explain the procedure for determining the stiffness modulus in step *i* + 1. The pavement layer moduli have been denoted as: *E*_1,i_, *E*_2,i_, *E*_3,i_—stiffness modulus of the asphalt layer, aggregate base course, and subgrade respectively in *i*-th step. In general, the stiffness modulus of *j*-th layer in *i*-th step is denoted as *E*_j,i_.

The calculated deflection of the pavement at the *k* measurement points in *i*-th step was denoted by *u*_o,k,i_.

The Secant Method (SM) is described further. To determine the value of the stiffness modulus in step *i* + 1, the relative changes in deflection values resulting from the modification of the value of elasticity modulus have been used. They were determined by calculations with changed modulus values—separately with increased modulus of elasticity for each of the three layers. Relative changes of deflection in *k*-th measurement points in *i*-th step with the stiffness modulus of *j*-th layer increased by the assumed size Δ*E*_j_ were denoted as *s*_k,j,i_. For example, the value of this change for the first layer is given in Equation (1):

(1) $$s_{k,1,i}=\frac{u_{o,k,i}(E_{1,i}+\Delta E_{1,i},E_{2,i},E_{3,i})-u_{o,k}{}_{,i}(E_{1,i},E_{2,i},E_{3,i})}{\Delta E_{1,i}}$$

where the symbol u_o,k,I_(*E*_1,i_, *E*_2,i_, *E*_3,i_) indicates the calculated deflection at *k*-th measurement point for the modulus values *E*_1,i_, *E*_2,i_, *E*_3_,_i_ of asphalt layer, aggregate base course, and subgrade, respectively. From the system of Equation (2), the values that modify the stiffness moduli of the structure layer were calculated. The system of Equation (2) is overdetermined; thus, the vector of unknowns was determined by the method of least squares. The vector values of unknowns are denoted by the following symbols: Δ*E*_1,i_, Δ*E*_2,i_, Δ*E*_3,i_. They were calculated on the assumption that their increment will have (due to their small values) a proportional effect on the change in the deflection value, such as adding the values Δ*E*_1,i_, Δ*E*_2,i_, Δ*E*_3,i_ when calculating the coefficients *s*_1,1,i_, *s*_1,2,i_,…, *s*_7,3,i_:

(2) $$s_{k,j,i}\Delta E_{j,i}=\Delta u_{k,i}$$

where Δ*u*_k,i_ = *u*_o,k,i_ − *u*_m,k_; *u*_o,k,i_—deflection calculated at *k*-th measurement point in *i*-th step; *u*_m,k_—deflection measured at *k*-th measurement points.

It was observed that the presented method of determining the pavement stiffness modulus is quickly convergent. The values of modulus changes used to determine the parameters *s*_k,1,i_ were ΔE_1,I_ = 500 MPa, ΔE_2,i_ = 50 MPa, ΔE_3,i_ = 10 MPa.

An important element of backcalculations is a function that determines the differences between measured displacements (or calculated but treated as measured in computer simulations) and those obtained from backcalculations. The root mean square difference (RMS) was used, determined by Equation (3) (another proposition can be area value with correction factor—AVCF described in the paper [29]):

(3) $$e_{\mathrm{RMS}}=\sqrt{\sum_{i}^{K}\frac{{\left(u_{m,}{}_{k}-u_{o,k}\right)}^{2}}{K}}$$

where u_m,k_—deflection measured at the *k*-*th* measuring point of a given deflection basin (µm), *u*_o,k_ = *u*_o,k,i_—deflection calculated at that point (µm), at the last *i*-th step, *K*—number of measuring points.

The value of eRMS equal to 0.5 µm has been assumed as the condition for completing the calculations.

### 2.2. Determination of Deflections and Strain

Obtaining the deflection values of road pavement for the assumed mechanical model of pavement and its load is crucial in the process of backcalculation. To perform calculations, the following (presented in Table 1) parameters of the pavement layers were established. The value of Poisson’s ratio was taken from [6].

**Figure 1:**
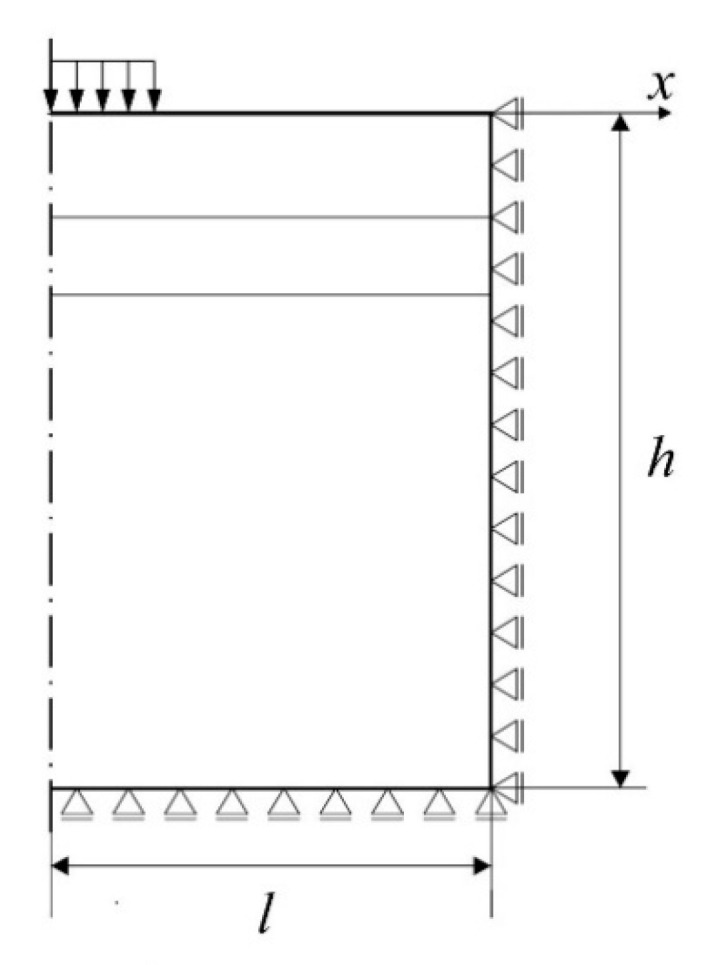
Section of the axisymmetric cylindrical area of a pavement modeled with the use of FEM.

The calculations were conducted using FEM in the Simulia Abaqus software (software version 6.14, Dassault Systemes, Vélizy-Villacoublay, France). A time-varying load was applied, described by the function *P*(*t*) = *P* sin(π*t*/*T*), which at *T* = 0.06 s simulates for *t* = 0.03 s the FWD action. The load is distributed evenly over a circular surface with a radius of *r* = 0.1368 m and a maximum intensity of *p* = 850 kPa and a resultant maximum force of *P* = 50 kN (*P*o = πr^2^ *p*). An axisymmetric model of a cylindrical pavement of such a size that the wave does not rebound from the edge of the domain in the case of *t* = *T*/2 for dynamic calculations (the depth *h* = 8 m and radius *l* = 9 m of this cylindrical area were assumed) was used. To obtain the strains, a static calculation model was assumed, in this case the load was equal to 50 kN and constant over time. The geometric model used in the pavement calculation and the assumed boundary conditions of the cylindrical area are presented in Figure 1.

**Figure 2:**
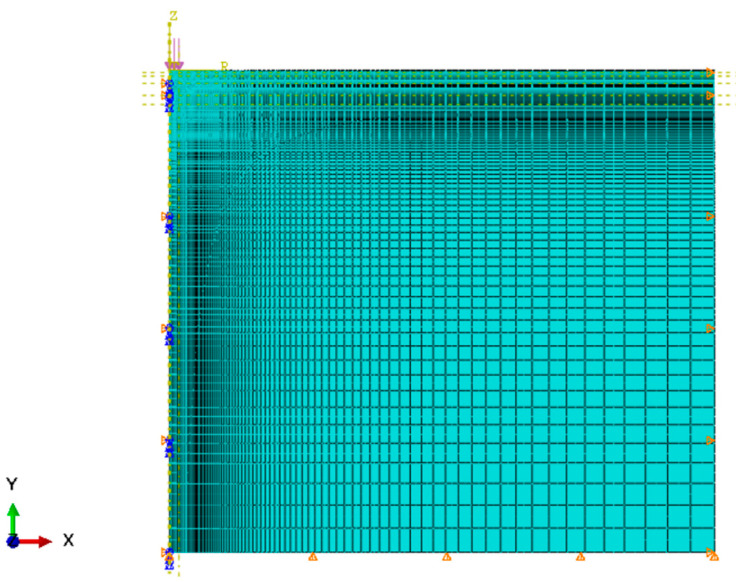
Finite element method mesh.

Rectangular finite elements with square shape functions and reduced integration CAX8R [36] have been applied. The FEM mesh provides sufficient accuracy for calculations—it is presented in Figure 2. The mesh size of 1.0 × 1.0 cm was used in the stress concentration area, the maximum values of the mesh dimension are 40.0 cm × 40.0 cm, additionally, the convergence of the results obtained for the proposed finite element mesh was checked—the mesh in total consists of 7820 elements.

### 2.3. Determination of Durability

The method from Mechanistic-Empirical Pavement Design Guide 2008 [37] was used to assess the durability of the pavement to compare the fatigue life for different stiffnesses. The calculated durability at the occurrence of fatigue cracks by bottom-up fatigue cracking (for established percentage damage of total lane area) is given by Equation (4):

(4) $$N_{f}=D_{FC}N_{\mathrm{ph}}N_{m}$$

(5) $$N_{\mathrm{ph}}=12.8828\cdot 10^{M},\;M=4.84\times \left(\frac{V_{a}}{V_{a}+V_{v}}-0.69\right)$$

(6) $$N_{m}=k_{1}(10^{-6})\;{\left(\frac{1}{\varepsilon_{h}}\right)}^{3.9492}{\left(\frac{1}{S_{a}^{}}\right)}^{1.281},k_{1}=\frac{1}{0.01+\frac{12}{1+e^{\left(15.676-1.1097\cdot h_{\mathrm{ac}}\right)}}}$$

(7) $$D_{FC}=\frac{1}{100}10^{\left[-C_{1}C_{1}^{\prime }+\ln\left(\frac{100}{FC}-1\right)\right]\frac{1}{C_{2}C_{2}^{\prime }}}$$

(8) $$C_{1}=1.0,\;C_{1}^{\prime }=-2C_{2}^{\prime },\;C_{2}=1.0,\;C_{2}^{\prime }=-2.40874-39.748{\left(1+\frac{h_{\mathrm{ac}}}{2.54}\right)}^{-2.856}$$

where *N*_f_—fatigue life (number of standard axles); *S*_a_—stiffness modulus of the lowest asphalt layer (MPa), in which cracks initiate; *ɛ*_h_—maximum tensile strain (horizontal) at the bottom of the asphalt layer in which cracks initiate; *v*_a_—asphalt content by volume in the mixture (% *v/v*) in the layer where the cracks initiate—assumed to be 11.1%; *v*_v_—free space volume content of the mixture (% *v/v*) in the layer where the cracks initiate—assumed to be 4.6%; *k*_1_—parameter determined in the calibration process of Equation (4), depending on the total thickness of the asphalt layer in case of cracks from bottom to top; *h*_ac_—total thickness of asphalt layer (cm); *D_FC_*—total fatigue damage on the surface at the occurrence of fatigue cracks on a percentage of the total lane area FC; and *C*_1_, *C*_1_’, *C*_2_, *C*_2_’—calibration factors.

The damage percentage of the total lane area (FC) was set to be equal to 5% and 15% in the moment of losing bearing capacity.

Equation (9) was used to calculate the durability due to the structural deformations:

(9) $$N_{d}={\left(\frac{k}{\varepsilon_{v}}\right)}^{1/m}$$

where *N*_d_—number of calculation axes due to permanent deformation; *k* = 1.05 × 10^−2^, *m* = 0.223 [38], *ε*_v_—maximum compressive strain in the natural subgrade.

### 2.4. Consideration of Measurement Error

In the current study, backcalculations were conducted for a surface deflection basin—the so-called measured deflection—with values: 280 µm, 252 µm, 236 µm, 189 µm, 150 µm, 119 µm, and 96 µm at subsequent measurement points. These values correspond to the deflections obtained for the model used with the asphalt layer, aggregate base course, and subbase stiffness moduli of 7356.1 MPa, 367.0 MPa, and 80.1 MPa respectively.

The influence of measurement errors on the calculation results has been analyzed. Four variants of measurement errors for deflections were considered:

- the normal distribution of errors is assumed with zero mean value and a standard deviation of the error equal to (2 µm + 0.01 u_i_)/3 (2 µm + 0.01 u_i_—value of maximum error comes from [39])—error calculated separately for each *i*-th measurement point of a given basin—variant denoted as D1S;
- the normal distribution of errors is assumed with zero mean value and a standard deviation of the error equal to (2 µm + 0.01 u_i_)—error calculated separately for each *i*-th measurement point of a given basin—variant denoted as D1B;
- the normal distribution of errors is assumed with zero mean value and a standard deviation of error equal to (2 µm + 0.01 u_1_)/3—error was firstly calculated for deflection in the load axis; the error for deflection in *i*-th measurement point is equal to the ratio of deflections u_i_/u_1_ multiplied by error in a first measurement point—variant denoted as D2S;
- the normal distribution of errors is assumed with zero mean value and a standard deviation of error equal to (2 µm + 0.01 u_1_)—error was firstly calculated for deflection in the load axis; the error for deflection in *i*-th measurement point is equal to the ratio of deflections u_i_/u_1_ multiplied by the error in a first measurement point—variant denoted as D2B.

For each error variant, calculations have been conducted in 100 cases, each time with a change of the measured deflection by a randomly selected value. The influence of deflection dispersion on the dispersion of the obtained values of material parameters, key values of strain, and on the values of fatigue life of the pavement for stiffness modulus was analyzed. The values of the variation coefficient *cv* (ratio of standard deviation to mean value) for deflections and for the resultant values (stiffness moduli, key strain, and fatigue life) were compared.

## 3. Results

### 3.1. Modulus of Stiffness of Pavement Layer

In this study, 100 backcalculations were conducted for each of the four variants of measurement error. In each case, the value of error was randomized, which was added to the simulation of real deflections. Backcalculations were then conducted in the subsequent steps of the iterative calculation process for such obtained deflections. In the last step (after fulfilling the condition of completing backcalculations), the values of asphalt layer, aggregate base course, and subgrade stiffness moduli were determined—denoted, respectively, as *E*_1_, *E*_2_, *E*_3_. The condition for ending the calculation is the value of root mean square error lower than 0.5 µm or not decreasing the errors in subsequent calculation steps. It was analysed how the coefficient of variation for the first deflection *cv*(*u*_1_) and the mean coefficient of variation of all seven deflections *cv*(*u*_av_) affects the coefficient of variation of the values of asphalt layer *cv*(*E*_1_), aggregate base course *cv*(*E*_2_) and subgrade *cv*(*E*_3_) stiffness moduli.

Table 2 presents the results of calculations for the analysed variants of errors in deflection measurements. For D1 variant, the relatively small measurement error of deflection resulted in significant errors in the asphalt layer and aggregate base course stiffness modulus. For example, for a variant with the coefficient of variation *cv*(*u*_av_) equal to 0.74 %, a value of *cv*(*E*_1_) = 9.18% and *cv*(*E*_2_) = 27.37% was obtained. A much smaller dispersion of the value of stiffness moduli occurred for the subgrade—coefficient of variation *cv*(*E*_3_) is only 1.92%. Increasing the dispersion of deflections within variant D1 adequately increases the error of the determined stiffness moduli.

In the case of D2 variants, similar input data dispersion as in D1 variants caused smaller dispersion of the values of pavement layer stiffness moduli. For D2S variant, the coefficient of variation for asphalt layer, aggregate base course, and subgrade was *cv*(*E*_1_) = 0.72%, *cv*(*E*_2_) = 0.24% and *cv*(*E*_3_) = 0.76% respectively. This corresponds to the order of magnitude of the coefficient of variation *cv*(*u*_1_) and *cv*(*u*_av_).

### 3.2. Strain and Fatigue Life

Using the obtained pavement stiffness moduli, the key strains necessary to assess the fatigue life of road pavement were calculated: tensile strain at the bottom of the asphalt layer (ε_h_) and the maximum compressive strain in the ground (ε_y_). Table 3 presents the values of the coefficient of variation for deflection and the coefficient of variation of tensile strain *cv*(ε_h_) and the coefficient of variation of compressive strain *cv*(ε_v_).

In the case of D2 error variant, the dispersion of strain values is of similar order of magnitude as the dispersion of deflection values—for D2S it was *cv*(*u*_av_) = 0.59%, *cv*(ε_h_) = 0.61% and *cv*(ε_v_) = 0.7%. Higher dispersion of key strains was obtained for D1S variant—1.52% for tensile strain and 2.8% for compressive strains. Nevertheless, it should be noted that these values were significantly lower than the coefficient of variation of stiffness modulus for D1S variant, which indicates that different variants of the stiffness modulus values imply similar values for key strains.

The pavement fatigue life was calculated due to fatigue cracks and permanent vertical deformation based on the obtained strain values. The dispersion of input deflection values and the dispersion of calculated durability values were compared—in relation to fatigue cracking of the pavement at 5% and 15% surface damage, and in relation to permanent subgrade deformation. The comparison results are presented in Table 4.

The value of durability coefficient of variation was greater than the value of strain coefficient of variation. This is a consequence of the highly monotonous dependence of the durability on the key strains in Equations (4) and (5). In the case of variant D2, the dispersion of durability results again was much smaller. For variant D2S, the coefficient *cv*(*N_f_*_15%_) and *cv*(*N_f_*_5%_) (i.e., 15% and 5% of the cracked area respectively) was 1.52% and *cv*(*N*_d_) was 3.16%. The coefficient of variation in the case of variant D1S was equal to 10.20% for fatigue cracking and 13.91% for permanent deformation, respectively.

## 4. Discussion

Firstly, it is worthwhile noting that there was a large difference in the dispersion of the values of the layer stiffness moduli between D1 and D2 variants. Even in the case of deflection variation coefficients that were similar, different dispersion values of the results for both variants were obtained. For example, for D1S variant it was *cv*(*E*_1_) = 9.81%, *cv*(*E*_2_) = 27.27%, *cv*(*E*_3_) = 1.92% and for variant D2S: *cv*(*E*_1_) = 0.72%, *cv*(*E*_2_) = 0.24%, *cv*(*E*_3_) = 0.76%. The difference between variants was caused by the fact that in the case of D2 variant the assumed error did not change the shape of the deflection basin. Randomization of deflections was carried out once for the deflection in the load axis, the remaining differences were calculated proportionally to the deflections at further measuring points. In the case of variant D1, for each measuring point, the error was randomized independently, which changed the shape of the deflection basin. The change of shape affected the proportions of pavement moduli.

**Figure 3:**
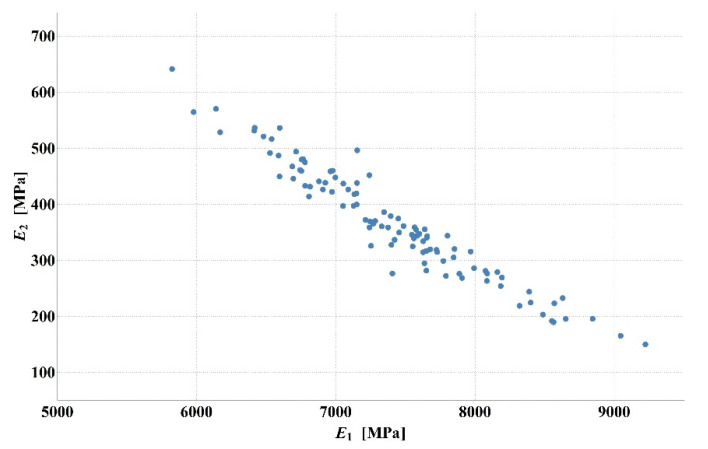
Values of asphalt layer and aggregate base course stiffness moduli for D1S variant of measurement error.

**Figure 4:**
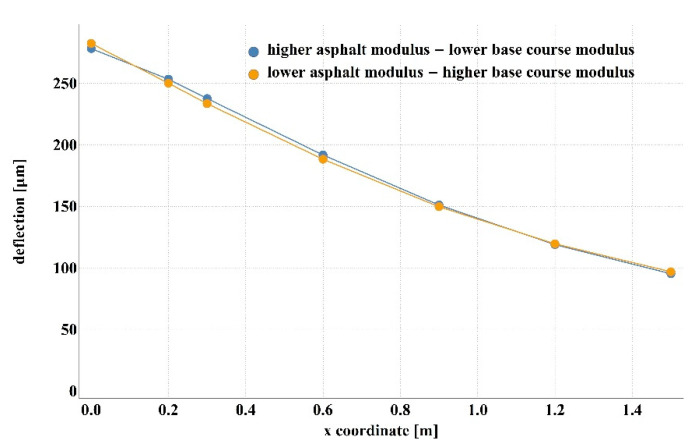
Deflection basin for the case with higher asphalt layer modulus—lower aggregate base course modulus and lower asphalt layer modulus—higher aggregate base course modulus.

**Figure 5:**
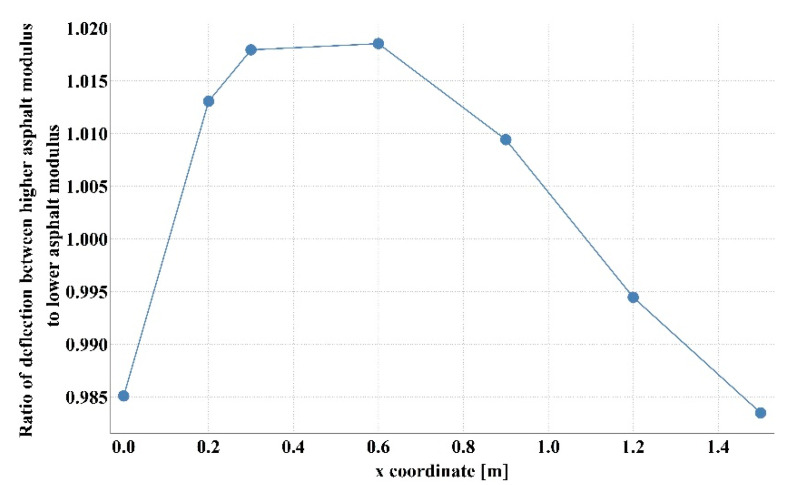
Ratio of displacement for the case with higher asphalt layer modulus—lower aggregate base course modulus to lower asphalt layer modulus—higher aggregate base course modulus.

Detailed analysis of the obtained values of stiffness moduli for D1S variant demonstrated that the main difference between the results consisted in a higher than average value of asphalt layer modulus with lower value of the base modulus or vice versa (smaller *E*_1_, larger *E*_2_). The results of 100 calculations for D1S variant are presented in Figure 3. The dispersion of stiffness modulus values was high with relatively small changes in the shape of the deflection basin. Such measurement errors can occur in real-time measurements. This may lead to incorrect parameter identification. It should be added that in the case of steeper deflection basin, higher values of the aggregate base course stiffness modulus and lower values of the asphalt layer stiffness modulus were obtained. In contrast, for a flatter deflection basin, lower values of the aggregate base course modulus and higher values of the asphalt layer modulus were observed. Figure 4 shows the deflection basin obtained for the values of moduli: *E*_1_ = 9223.51 MPa, *E*_2_ = 150.03 MPa, *E*_3_ = 81.34 MPa and for the values of moduli: *E*_1_ = 5823.60.51 MPa, *E*_2_ = 641.26 MPa, *E*_3_ = 77.64 MPa as an example of the results for the case of higher asphalt modulus—lower base course modulus and the case of lower asphalt modulus—higher base course modulus. Figure 5 presents the ratio of displacement in certain measurement points for the two analysed cases. The observed relation is due to the fact that the closer the displacements are measured to the load axis, the greater the influence of the stiffness of the upper pavement layers on the deflections.

**Figure 6:**
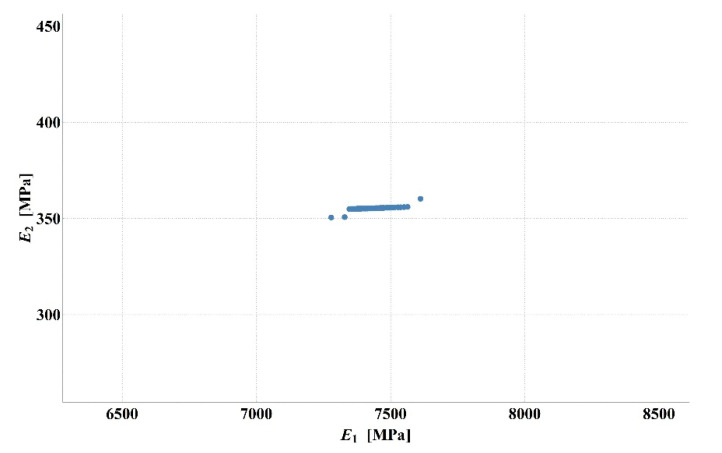
Values of asphalt layer and aggregate base course stiffness moduli for D2S variant of measurement error.

In the case of D2S variant of the measurement error—simulating a systematic error, analogous for all measurement points—the dispersion of values was smaller, and for larger values of the asphalt layer modulus, a larger aggregate base course modulus was obtained. This is illustrated in Figure 6.

**Figure 7:**
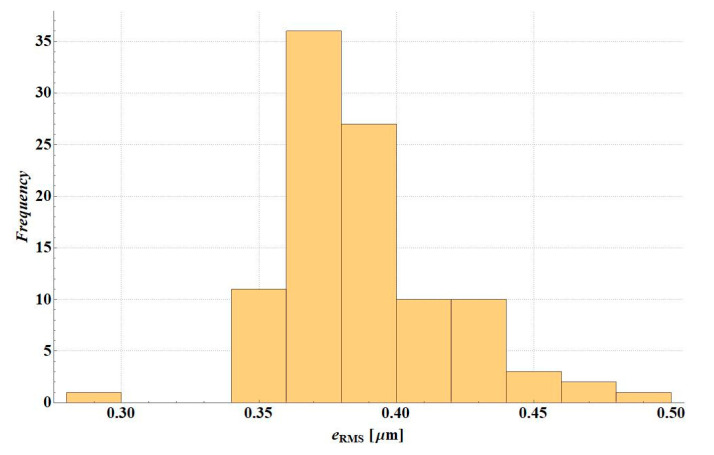
*e*_RMS_ error histogram for D2S variant.

**Figure 8:**
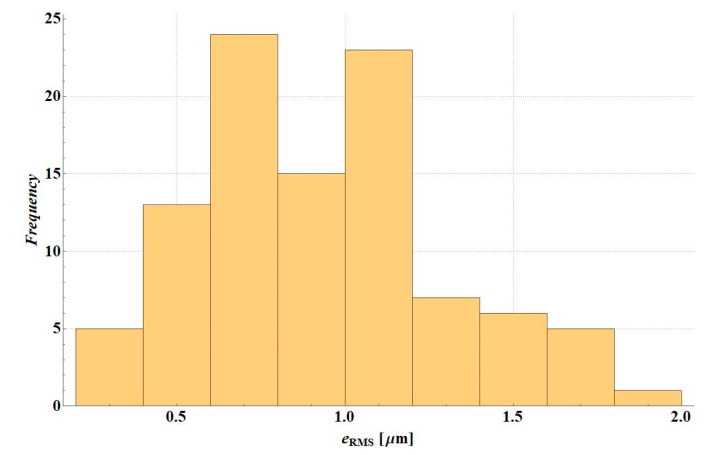
*e*_RMS_ error histogram for D1S variant.

The *e*_RMS_ error was analysed in simulation variants of the deflection measurement error. Figure 7 presents the error histogram for variant D2S, Figure 8 illustrates the error histogram for variant D1S. In the first case, the error was small—the value is lower than 0.5 µm. It resulted from the fact that in this variant the shape of the deflection basin was not changed. After a random error, it is still physical and material parameters can be selected to obtain consistent deflections. For D1S error variant, in many cases of random deflection measurement errors, no solution could be found, i.e., a system of stiffness modulus values for which the deflection measurement error would be less than 0.5 µm (applied criterion for the end of backcalculations).

**Figure 9:**
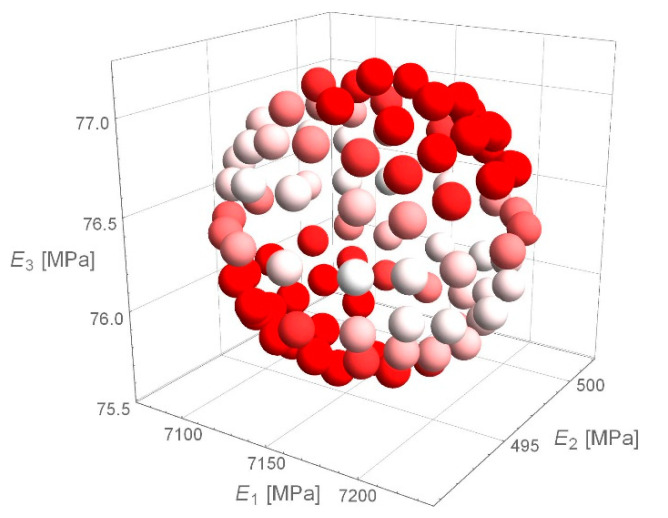
*e*_RMS_ error for individual combinations of material parameters—spherical domain.

**Figure 10:**
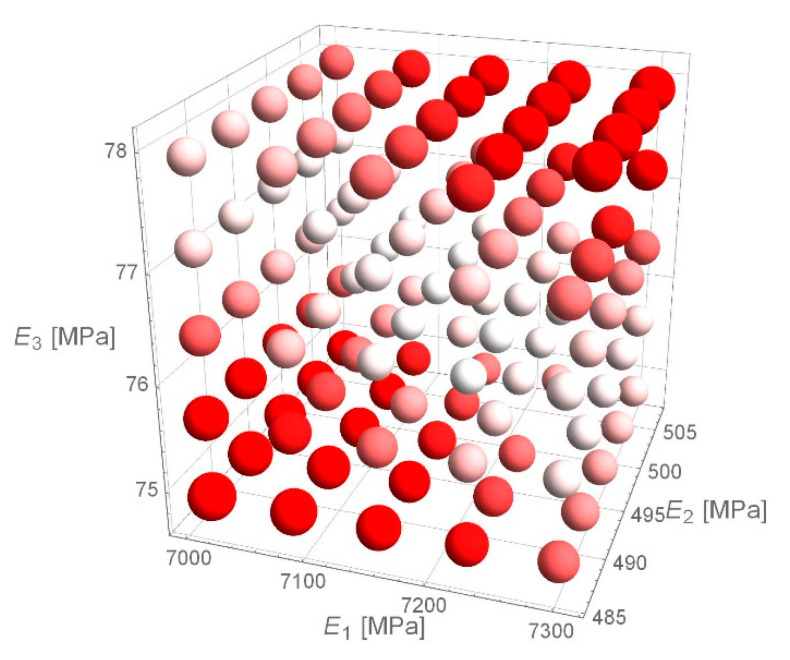
*e*_RMS_ error for individual combinations of material parameters—cubic domain.

**Figure 11:**
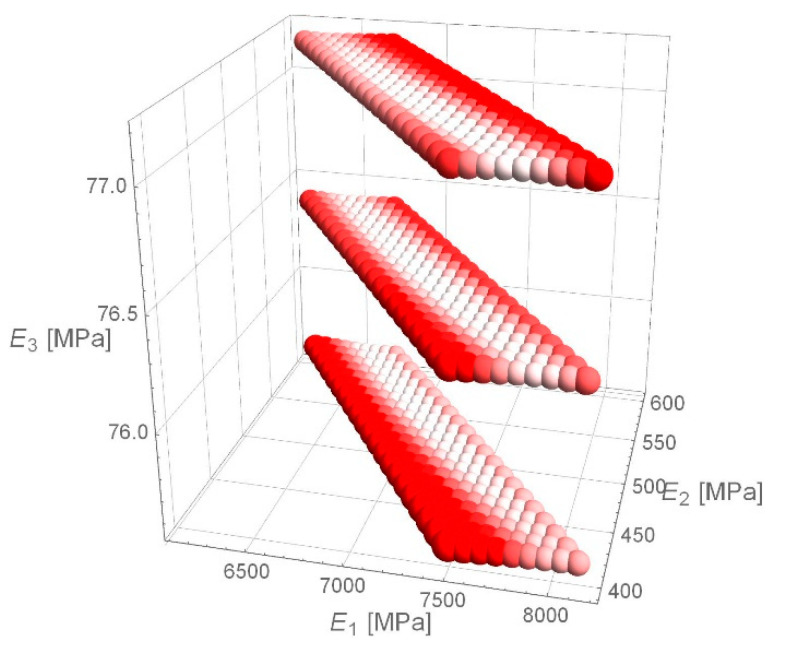
*e*_RMS_ error for individual combinations of material parameters—cuboidal domain.

Due to the *e*_RMS_ error histogram for D1S variant, it was verified whether the received values of stiffness moduli achieve the minimum of *e*_RMS_ function. The paper presents an example of calculations for the deflection basin for which *the e_RMS_* error equals to 1.8 µm was obtained. The values around the determined values of stiffness moduli obtained as a result of backcalculations were verified. The spherical domain around the fixed values of stiffness modulus (Figure 9), the cubic domain (Figure 10) and the cuboidal domain (Figure 11) were searched—the white values on drawings indicate the lower value of *e*_RMS_ error, the more intense the red colour, the higher the error value is. For a point obtained from backcalculations, the value of *e*_RMS_ error is less than for any point in its neighbourhood.

For D1 variant, the dispersion of strain was significantly smaller than the dispersion of material stiffness moduli. The dispersion of stiffness moduli in D1 case resulted from a change in the shape of the deflection basin. This caused changes in the proportions of asphalt layer and aggregate base course stiffness moduli. It should be noted that different combinations of layer parameter values may give similar values of strains, which can be observed in the obtained calculation results.

Increasing dispersion of the calculated durability value resulted from the strong monotony of its dependence on the value of key strain in the equation for durability—a relatively small change in horizontal strain at the bottom of the asphalt layer implied a large change in fatigue life. In addition, there was a stiffness modulus value in the fatigue life equation, which increased the error of obtained value—regardless of the strain determination error.

## 5. Conclusions

In conclusion, attention should be drawn to the high sensitivity of the values of stiffness modulus, strains and obtained fatigue life to the measurement error of deflection when every deflection error is random, which changes the shape of the deflection basin (case D1). For a deflection coefficient of variation of 0.74%, the coefficient of variation for moduli of asphalt layer, aggregate base course and subgrade equal to 9.18%, 27.37%, and 1.92%, respectively, was obtained. A small measurement error leads to a large moduli of asphalt layer and moduli of aggregate base course determination error. Using the obtained moduli values, key strains were computed—coefficient of variation of maximum tensile strain (horizontal) at the bottom of the asphalt layer was equal to 1.52% and the coefficient of variation of maximum compressive strain in the natural subgrade was equal to 2.89%. The variability for strain is significantly less than for modulus. For a similar deflection basin, similar strain values are obtained. Durability coefficient of variation of 10.20% was obtained. In other words, a small measurement error leads to a large durability determination error.

The high sensitivity was mainly caused by the similar deflection results obtained for completely different combinations of the values of asphalt layer and aggregate base course stiffness modulus. The deflection basin for the reduced-value asphalt layer modulus and the increased-value base layer modulus differed, albeit only slightly in shape, from the deflection basin for the increased-value asphalt layer modulus and the reduced value of the aggregate base course modulus. However, the strains in both cases were slightly different, and the structure of the fatigue life equation multiplied the differences for pavements with very similar deflection basins.

In case of errors in measuring deflections of a similar, appropriately proportional size that did not change the shape of the deflection basin (case D2), the identification errors of stiffness modulus, strain and durability were small. They had the same order of magnitude as the errors of deflection. Deflection coefficient of variation is equal to 0.74%. Coefficient of variation for the moduli of asphalt layer is equal to 0.72%, for the moduli of aggregate base course is equal to 0.24%, and for moduli of subgrade is equal to 0.76%. Strains also have similar values for all analysed deflection basins—coefficient of variation of maximum tensile strain (horizontal) at the bottom of the asphalt layer is equal to 0.61% and the coefficient of variation of maximum compressive strain in the natural subgrade is equal to 0.70%. It leads to a coefficient of variation for durability equal to 1.52%.

The obtained results draw attention to the influence of measurement uncertainties on the backcalculation results. This impact should be considered when determining the value of the modulus of elasticity. Furthermore, the deflection measurement error values should be limited. When the errors are random in nature, this can be achieved by taking several measurements in one road section and calculating the average. Moreover, with known measurement accuracy, it would be possible to create envelopes of the deflection basin to carry out several sets of backcalculation and give the results of modulus values as intervals.

## Figures and Tables

**Table 1:** Geometrical and material parameters of road structure

#	Layer	*h* (cm)	*ρ* (kg/m^3^)	*ν* (m/m)
1.	Asphalt layer	22	2610	0.3
2.	Aggregate base course	20	2250	0.3
3.	Subgrade	∞	1800	0.35

**Table 2:** Variation coefficient of deflection and stiffness modulus.

#	Variant	$cv(u_{1})\;(\%)$	$cv(u_{\mathrm{av}})\;(\%)$	$cv(E_{1})\;(\%)$	$cv(E_{2})\;(\%)$	$cv(E_{3})\;(\%)$
1.	D1S	0.59	0.74	9.18	27.37	1.92
2.	D1B	1.75	2.13	21.77	55.16	4.62
3.	D2S	0.59	0.59	0.72	0.24	0.76
4.	D2B	1.75	1.75	2.14	1.00	2.26

**Table 3:** Variation coefficient of deflection values and key strains

#	Variant	$cv(u_{1})\;(\%)$	$cv(u_{\mathrm{av}})\;(\%)$	$cv(\varepsilon_{h})\;(\%)$	$cv(\varepsilon_{v})\;(\%)$
1.	D1S	0.59	0.74	1.52	2.80
2.	D1B	1.75	2.13	4.80	8.97
3.	D2S	0.59	0.59	0.61	0.70
4.	D2B	1.75	1.75	1.92	2.11

**Table 4:** Variation coefficient of deflection values and design durability

	Variant	$cv(u_{1})\;(\%)$	$cv(u_{\mathrm{av}})\;(\%)$	$cv(N_{f\;5\%})\;(\%)$	$cv(N_{f\;15\%})\;(\%)$	$cv(N_{d})\;(\%)$
1.	D1S	0.59	0.74	10.20	10.20	13.91
2.	D1B	1.75	2.13	26.12	26.12	56.32
3.	D2S	0.59	0.59	1.52	1.52	3.16
4.	D2B	1.75	1.75	4.86	4.86	9.49

## Data Availability

All data included in this study are available upon request by contact with the corresponding author.
